# Competing ultrafast photoinduced electron transfer and intersystem crossing of [Re(CO)$_3$(Dmp)(His124)(Trp122)]$^+$ in *Pseudomonas aeruginosa* azurin: a nonadiabatic dynamics study

**DOI:** 10.1007/s00214-020-2555-6

**Published:** 2020-03-17

**Authors:** Sebastian Mai, Maximilian F. S. J. Menger, Marco Marazzi, Dario L. Stolba, Antonio Monari, Leticia González

**Affiliations:** 1 Institute of Theoretical Chemistry, Faculty of Chemistry, University of Vienna, Währinger Str. 17, 1090 Vienna, Austria; 2 Present Address: Photonics Institute, Vienna University of Technology, Gußhausstr. 27–29, 1040 Vienna, Austria; 3 Present Address: Zernike Institute for Advanced Materials, Faculty of Science and Engineering, University of Groningen, Nijenborgh 4, 9747 AG Groningen, The Netherlands; 4 Department of Analytical Chemistry, Physical Chemistry and Chemical Engineering, Universidad de Alcalá, Ctra. Madrid-Barcelona Km. 33,600, 28871 Alcalá de Henares, Madrid Spain; 5 Chemical Research Institute “Andrés M. del Río” (IQAR), Universidad de Alcalá, 28871 Alcalá de Henares, Madrid Spain; 6 Université de Lorraine and CNRS, LPTC UMR, 7019 Nancy, France

**Keywords:** Protein electron transfer, Quantum chemistry, Nonadiabatic dynamics, Intersystem crossing

## Abstract

We present a computational study of sub-picosecond nonadiabatic dynamics in a rhenium complex coupled electronically to a tryptophan (Trp) side chain of *Pseudomonas aeruginosa* azurin, a prototypical protein used in the study of electron transfer in proteins. To gain a comprehensive understanding of the photoinduced processes in this system, we have carried out vertical excitation calculations at the TDDFT level of theory as well as nonadiabatic dynamics simulations using the surface hopping including arbitrary couplings (SHARC) method coupled to potential energy surfaces represented with a linear vibronic coupling model. The results show that the initial photoexcitation populates both singlet metal-to-ligand charge transfer (MLCT) and singlet charge-separated (CS) states, where in the latter an electron was transferred from the Trp amino acid to the complex. Subsequently, a complex mechanism of simultaneous intersystem crossing and electron transfer leads to the sub-picosecond population of triplet MLCT and triplet CS states. These results confirm the assignment of the sub-ps time constants of previous experimental studies and constitute the first computational evidence for the ultrafast formation of the charge-separated states in Re-sensitized azurin.

## Introduction

Photoinduced electron transfer (PET) in complex biological, multi-chromophoric materials—such as proteins—is a phenomenon of the utmost importance in chemistry and biology [1–3]. PET is at the base of solar energy conversion and storage, most notably via the photosynthetic chain processes in plants and bacteria. Even if extremely widespread, and affecting all living organisms, photosynthesis remains a very complex and fascinating process, although some of its fundamental aspects are not yet completely understood at the molecular and electronic scale. One aspect that requires further study is how long-range PET processes in photosynthesis achieve their very high efficiency [4, 5], even though there are several other excited-state decay mechanisms—such as non-radiative relaxation, luminescence, or photoreactivity—that would disrupt any PET. Previous research [6] has shown that the electrostatic and mechanical effects of the macromolecular environment are fundamental to increasing the PET efficiency, e.g., by aligning the chromophores and by favorably adjusting the shape of the relevant excited-state potential energy surfaces (PESs).

**Fig. 1:**
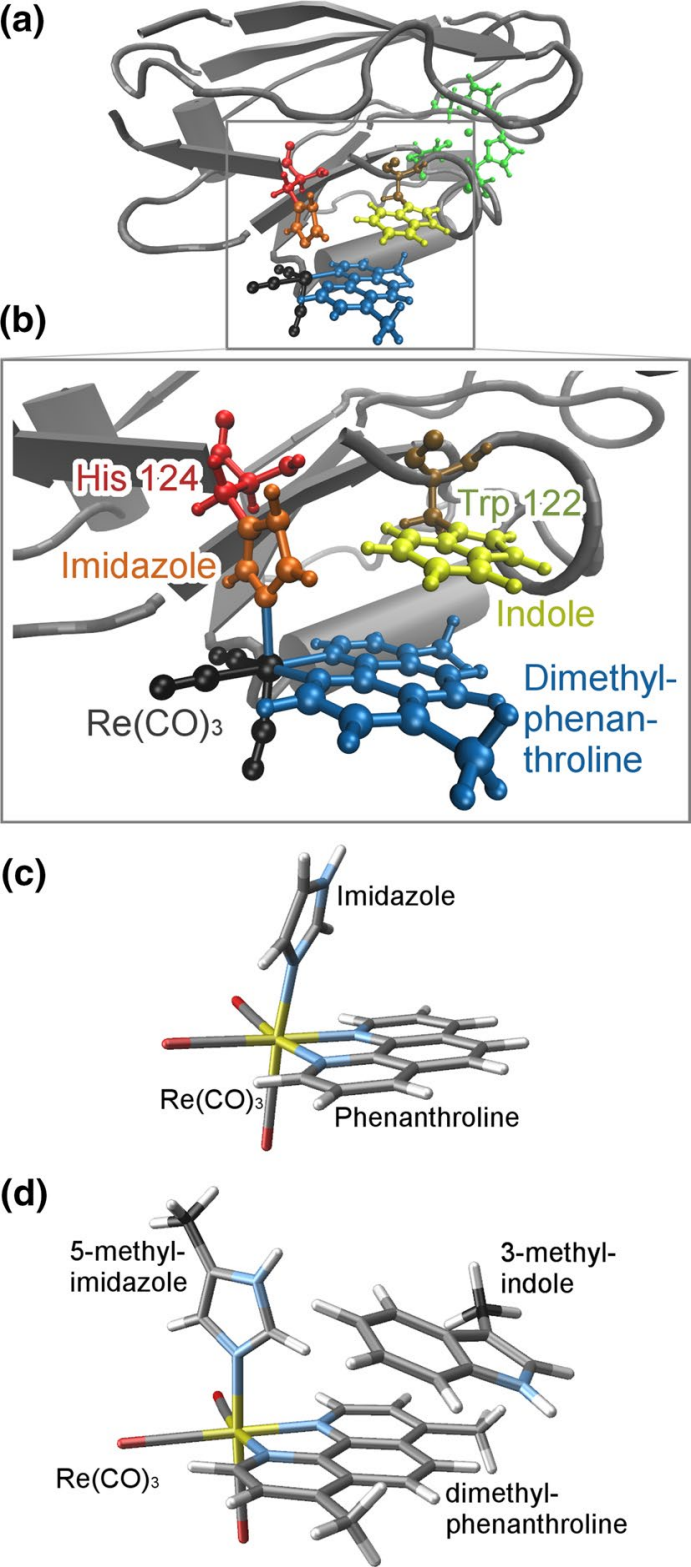
**a** The azurin protein (gray) with His124, Trp122, the Re complex, and the Cu center (green) highlighted. **b** Zoom on the moieties studied here: Trp122 in brown/yellow (indole in yellow), His124 in red/orange (imidazole in orange), $\hbox {Re(CO)}_{3}$ group in black, and the Dmp ligand in blue. **c** The “small system” [Re(CO)$_3$(phenanthroline)(imidazole)]$^+$. **d** The Trp-including model system studied here (anchor atoms in black)

Because of its importance and complexity, PET in proteins was the object of a number of experimental [2, 3, 7–9] and theoretical studies [10, 11]. In many cases, the sensitization of naturally occurring proteins with heavy metals complexes, including Re or Ru, has been pursued to facilitate its photophysical characterization. For example, the azurin protein of *Pseudomonas aeruginosa* has been sensitized with a Re organometallic complex, namely [Re(CO)$_3$(Dmp)(His)]$^+$ (Dmp $=$ 4,7-dimethylphenanthroline), as shown in Fig. 1a. The azurin protein is involved in the respiratory chains acting as an electron shuttle, thanks to the presence of a Cu center that can easily switch between the +II and +I oxidation states. The sensitization with the Re subunit has allowed to identify a fast photoinduced electron transfer upon visible light irradiation, with charge migration between the two metal centers, i.e., Cu and Re. Most notably, this process involves a very long-range charge transfer of approximatively 30 Å, happening in the ns time scale.

**Fig. 2:**
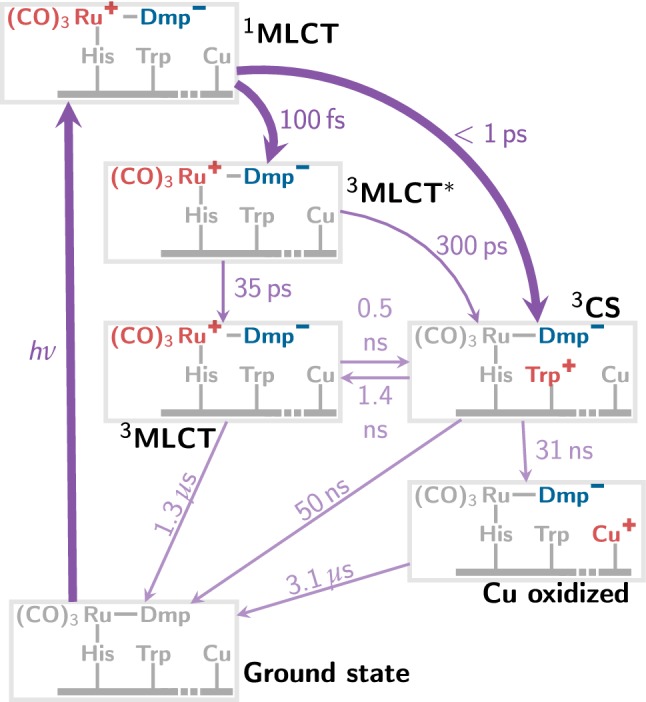
Scheme of the electron transfer processes occurring in [Re(Dmp)(CO)$_3$(His)]$^+$-sensitized *Pseudomonas aeruginosa* azurin after vertical excitation according to Ref. [9]

For the Re-complex-sensitized azurin, a multitude of different time-resolved spectroscopic experiments—including transient absorption [9], time-resolved infrared [8, 9, 12, 13], and time-resolved luminescence [9]—were published in the last decades. The bulk of this work was previously summarized into a complex multistep photocycle [9, 13], of which the important steps are shown in Fig. 2. After excitation, the first step involves ultrafast and efficient intersystem crossing (ISC) and hence the population of triplet metal-to-ligand charge transfer ($^{3}\hbox {MLCT}$) states, followed by the establishing of an equilibrium with a charge-separated (CS) state, that will in turn induce the electron transfer from the Cu center. The $^{3}\hbox {MLCT}{-}^{3}\hbox {CS}$ equilibrium was characterized by time constants in the ns range and is due to the favorable $\pi \hbox {-stacking}$ arrangement of the Dmp ligand of the Re complex with a proximal tryptophan (Trp) side chain that can act as an electron donor. The spatial proximity of the metal complex and the Trp residue is seen in Fig. 1b. Very interestingly, the spectroscopic results indicated that also a second ultrafast channel is present, leading to the direct population of $^{3}\hbox {CS}$ at the sub-picosecond time scale.

On the quest to gain a more detailed understanding of the different processes of this photocycle, several computational studies were carried out in the past, treating three different system sizes: (1) the “small system” including only the [Re(CO)$_3$(phenanthroline)(imidazole)]$^+$ complex (with or without solvent), (2) the “medium system” consisting of the Re complex attached to a His-Gly-Trp tripeptide (in solution), and (3) the “large system” encompassing the full azurin protein sensitized with the Re complex. The ultrafast dynamics of the small system (shown in Fig. 1c) was studied in detail with quantum chemistry [14–16], quantum dynamics [17–19], model-potential-based surface hopping [20], or QM/MM-based surface hopping [16, 21–23]. These studies have firmly established the importance of the environment effects on the absorption spectrum and the ultrafast ISC of [Re(CO)$_3$(phenanthroline)(imidazole)]$^+$.

In contrast to the small system, the photocycle of the medium and large systems—both of which include the Trp amino acid—has been investigated to a much smaller extent. To the best of our knowledge, the only study on that topic reports [6] classical molecular dynamics (MD) simulations, showing that only in the presence of the azurin scaffold the optimal $\pi \hbox {-stacking}$ of Dmp ligand and Trp is maintained. Using MD based on specifically parametrized force fields for $^{3}\hbox {MLCT}$ and $^{3}\hbox {CS}$ states and subsequent density functional theory/molecular mechanics (DFT/MM) calculations, it was also shown that the electrostatic field generated by the protein environment is essential in shaping the PESs of these states. The PES minima of both states exhibit similar energies, which leads to the above-mentioned MLCT–CS equilibrium that evolves on a ns time scale (see Fig. 2).

However, one very important aspect not tackled yet from a theoretical perspective is the role that the $^{3}\hbox {CS}$ state plays in the ultrafast (i.e., sub-ps) dynamics. According to spectroscopic studies [9, 13], the CS state is already partially populated within the first picosecond after excitation. It is nevertheless not clear to what extent the CS state is populated on this time scale, and what the underlying mechanism is—e.g., direct population transfer from $^{1}\hbox {MLCT}$, transfer via $^{1}\hbox {CS}$, direct photoexcitation of $^{1}\hbox {CS}$, or conversion of $^{3}\hbox {MLCT}$ to $^{3}\hbox {CS}$. Motivated by these questions, in this contribution we present the first nonadiabatic dynamics simulations of the coupled [Re(CO)$_3$(Dmp)(His)]$^+$ and Trp system (see Fig. 1d). Our simulations build upon the previously published molecular dynamics simulations [6] to identify the most important geometrical arrangement of the system and the mechanical constraints exerted by the protein scaffold. Based on these constraints and a thorough characterization of the excited-state manifold, we develop a linear vibronic coupling (LVC) model [24] of the PESs, which is subsequently employed in surface hopping including arbitrary coupling (SHARC) [25, 26] simulations of the nonadiabatic evolution of the system. The results show the complex nature of the ultrafast decay mechanisms of the Re-sensitized azurin and in particular the relevance of the direct $^{3}\hbox {CS}$ population especially at very short time.

## Computational details

### Molecular dynamics simulation

The initial geometry used in this study was extracted from an MM-MD simulation of Re-sensitized azurin in water, as presented elsewhere [6]. See Fig. 1a, b for depictions of the protein and the most important structural units. From the snapshots taken from this MD simulation, one was selected that was representative for the distance between the Re complex and the Trp residue. From this snapshot, we extracted the coordinates of [Re(CO)$_3$(Dmp)(His)]$^+$ and Trp. The His residue was simplified to a 5-methyl-imidazole, and the Trp was simplified to 3-methyl-indole, as shown in Fig. 1d. The carbon atoms of the two inserted methyl groups (highlighted in black in Fig. 1d) were fixed in the laboratory frame in order to replicate the constraining effect of the protein scaffold. For the extracted snapshot, the distance of these two carbon atoms was 7.98 Å.

### Ground-state level of theory

The prepared structure with the two fixed carbon atoms was subsequently optimized in the singlet ground state, and a frequency calculation was carried out, yielding all positive frequencies. These computations were performed at the B3LYP/6-31G* level of theory [27, 28], with the LANL2DZ effective core potential used for Re [29]. Dispersion was treated with the Grimme D3 correction (GD3) [30]. Solvent effects were approximately treated with the IEFPCM method [31], where we chose acetone ($\varepsilon =20.493$) as a compromise to describe the polarity of the surrounding water solvent and the less polar protein environment, as suggested elsewhere [32]. These calculations were performed with the Gaussian 09 [33] suite.

### Excited-state level of theory

At the optimized ground-state geometry, we conducted vertical excitation calculations within the linear response formulation of time-dependent density functional theory (TDDFT). For these calculations, the Amsterdam Density Functional (ADF) [34] suite was employed. We used the B3LYP functional [27] and the ZORA relativistic Hamiltonian [35] together with the ZORA-SVP basis set for all atoms, except for Re where we used ZORA-TZP [36]. Dispersion correction was included through the D3 Becke–Johnson scheme [37]. Solvent effects were described with the conductor-like screening model (COSMO) [38, 39], again with acetone as solvent ($\varepsilon _0=20.7, \varepsilon _\infty =1.8463$). “Good” quality settings were used for the Becke numerical integration grid [40], the ZlmFit Coulomb fit method [41], and the resolution of identity (RI) Hartree–Fock scheme [42].

Using the Tamm–Dancoff approximation (TDA) [43] and the adiabatic local density approximation [44], we computed 30 singlet states (ground state plus 29 excited states) and 30 triplet states. Spin–orbit couplings (SOCs) were computed with the spin–orbit perturbational approach implemented in ADF [45]. The charge transfer characters of these states were analyzed using the TheoDORE suite of programs [46, 47].

### LVC parametrization

In this work, we employ an analytical LVC model to describe the excited-state PESs of the [Re(Dmp)(CO)$_3$(His)]$^+$ plus Trp system. Details about the analytical LVC models employed within SHARC and the parametrization procedure are given in Refs. [24, 48]; hence, here we only provide a brief overview.

In the LVC approach, the diabatic PESs are approximated in terms of the ground-state PES $V_0$ and state-specific vibronic coupling terms ${\mathbf {W}}$:

1 $$\begin{aligned} {\mathbf {V}}=V_0{\mathbf {1}}+{\mathbf {W}}. \end{aligned}$$

The ground-state PES is described by a harmonic oscillator in a basis of mass–frequency-scaled normal mode coordinates $\{Q_i\}$:

2 $$\begin{aligned} V_0(\mathbf {Q})=\sum _i\frac{\hbar \omega _i}{2}Q_i^2, \end{aligned}$$

with the normal mode frequencies $\omega _i$. This PES is the basis for the PESs of all states, but for each state the PES is shifted by the diagonal terms

3 $$\begin{aligned} W_{nn}(\mathbf {Q})=\epsilon _n+\sum _i\kappa _i^{(n)}Q_i \end{aligned}$$

and the states are coupled via the off-diagonal terms:

4 $$\begin{aligned} W_{nm}(\mathbf {Q})=\sum _i\lambda _i^{(n,m)}Q_i. \end{aligned}$$

In these equations, $\epsilon _n$ are the vertical energy shifts, $\kappa _i^{(n)}$ the state-specific gradients in normal mode coordinates, and $\lambda _i^{(n,m)}$ are the linear vibronic coupling terms. The normal mode coordinates can be computed from the Cartesian coordinates via:

5 $$\begin{aligned} Q_i=\sqrt{\omega _i}{\hbar }\sum _\alpha K_{\alpha i}\sqrt{M_\alpha }\left( r_\alpha -r_\alpha ^{\mathrm {ref}}\right) , \end{aligned}$$

where ${\mathbf {K}}$ is the Cartesian-normal mode transformation matrix, $M_\alpha$ is the mass of atom $\alpha$, and $\mathbf {r}^{\mathrm {ref}}$ is the reference geometry.

To parametrize the LVC model, the parameters $r_\alpha ^{\mathrm {ref}}$, $\omega _i$ and $K_{\alpha i}$ were taken from the ground-state frequency calculation described above. For the excited-state parameters, we defined the diabatic basis to be identical to the adiabatic states at the reference geometry. Then, $\epsilon _n$ parameters were the vertical excitation energies at the reference geometry, and $\kappa _i^{(n)}$ parameters are obtained by transforming the gradients at this geometry into the normal mode basis. $\lambda _i^{(n,m)}$ parameters were obtained from wave function overlaps with slightly displaced geometries as described previously [48]. We used two-sided displacements with a step length of 0.05 in mass–frequency-weighted normal mode coordinates, and the overlaps were computed from wave functions truncated to 99.7% of the norm [49].

The original TDDFT calculations used for the parametrization encompassed 30 singlet ($S_0$ and 29 excited) states, 30 triplet states, and all 192 normal modes. In order to avoid different numerical problems with the simulations, this initial LVC model was subsequently truncated. First, low-frequency modes in the harmonic oscillators are prone to show very large nuclear displacements that (1) are unrealistic because such modes are often very anharmonic, and (2) lead to very large $\lambda _i^{(n,m)}Q_i$ contributions that lead to spurious coupling between all states. To mitigate these problems, we removed all normal modes with $\omega _i<300\,$ cm$^{-1}$, i.e., the lowest 34 normal modes (leaving 158 modes in the model). Second, some of the high-lying states showed extremely strong mixing that could not fully be resolved with the finite numerical precision of the calculation, leading to a large number of spuriously large $\lambda _i^{(n,m)}$ parameters. Hence, we removed these high-lying states ($S_{20}$ to $S_{29}$, $T_{16}$, $T_{18}$, $T_{20}$ to $T_{30}$), retaining 20 singlet states (including $S_0$) and 17 triplet states. Finally, we removed the SOCs of $S_0$ with the other states, as $S_0$ in DFT is not described on the same footing as the other states, which can create artificial $S_0$–triplet crossings where the system could erroneously relax to the ground state.

During the initial vertical excitation calculations and during the dynamics simulations, these parameters were used to compute all properties required by SHARC in the following way [24]. (1) The Cartesian geometry is transformed into normal mode coordinates. (2) The matrix ${\mathbf {V}}$ in Eq. (1) is computed from the parameters. (3) This matrix is diagonalized by ${\mathbf {V}}={\mathbf {U}}{\mathbf {E}}^{\mathrm {MCH}}{\mathbf {U}}^\dagger$ to switch from the diabatic basis (used to define the parameters) into the molecular Coulomb Hamiltonian (MCH) eigenbasis (i.e., adiabatic with respect to $\lambda _i^{(n,m)}$ parameters, but spin-free) [25] that is used as input for SHARC. (4) The diabatic dipole moment matrices and SOCs are transformed into the MCH basis using ${\mathbf {U}}$. (5) Gradients and nonadiabatic couplings are computed from ${\mathbf {U}}$ and the derivatives of ${\mathbf {V}}$ and back-transformed to the Cartesian representation. (6) Wave function overlaps $\langle \varPsi _i(t)|\varPsi _j(t+\Delta t)\rangle$ are computed from ${\mathbf {U}}(t)$ and ${\mathbf {U}}(t+\Delta t)$. These six steps produce all quantities (energies, SOCs, dipole moments, gradients, nonadiabatic couplings, overlaps) needed to run the SHARC–LVC simulations in full analogy to ab initio SHARC dynamics.

### Nonadiabatic dynamics simulations

1000 initial conditions for the SHARC simulations were sampled from the Wigner distribution of the ground-state harmonic oscillator that is defined by $V_0$ in Eq. (2). At each of these initial conditions, we performed a vertical excitation calculation as described above. From these 1000 calculations, we generated an absorption spectrum of the system. The initial excited states were determined stochastically based on the oscillator strengths [50] in the energy window 2.8–3.2 eV, the same that was used previously in the ab initio SHARC dynamics simulations of the small system [22]. The stochastic algorithm selected 200 initial conditions, where 6 started in $S_1$, 36 in $S_2$, 58 in $S_3$, 52 in $S_4$, 35 in $S_5$, 8 in $S_6$, 4 in $S_7$, and 1 in $S_8$ (all in the MCH representation).

The surface hopping dynamics simulations were performed with the SHARC approach [25], where the input data are in the MCH representation (as described above) and are on-the-fly transformed to the diagonal representation, in which the full Hamiltonian including the SOCs is diagonalized. All 200 initial conditions were propagated for 1000 fs using a 0.5 fs time step for the nuclear motion. The electronic wave function was propagated with a 0.02 fs step with the local diabatization procedure [25, 51]. To avoid overcoherence problems, an energy-based decoherence correction [52] was applied. Energy conservation during hops was achieved by rescaling the full velocity vector.

The nonadiabatic dynamics simulations were carried out with the SHARC2.0 suite of programs [25, 26] using its PySHARC driver [24] for the propagation. In order to further increase the computational efficiency of the PySHARC driver, for the present work we have implemented support for the Network common data format (NetCDF) [53] to reduce the size of the output data files. As file I/O represents a considerable fraction of the total computational time for the LVC–SHARC simulations, this file size reduction also leads to a significant speed-up of the simulations.

### Analysis

The most important result of the propagated trajectories is the evolution of the electronic wave function, which was analyzed in three complementary ways. First, we obtained the electronic populations in the MCH representation using a partial Wigner transform as described in Ref. [54]. Second, the electronic populations in the diabatic representation (i.e., the basis composed of the excited states at the reference geometry) were obtained with the same transformation technique. Third, the charge transfer character of the propagated electronic wave function was analyzed. Since there are no explicit wave functions in the LVC model, we obtained the charge transfer numbers of the propagated wave function $\Omega _{AB}^{\mathrm {el}}(t)$ by transforming the charge transfer numbers of the states that form the diabatic basis:

6 $$\begin{aligned} \Omega _{AB}^{\mathrm {el}}(t)=\sum _n\sum _m c^{{\mathrm {diab}}*}_n(t)c^{\mathrm {diab}}_m(t)\Omega _{AB}^{(n,m)}, \end{aligned}$$

where *A* and *B* are fragments of the molecule, $c^{\mathrm {diab}}$ are the electronic wave function coefficients in the diabatic basis, and $\Omega _{AB}^{(n,m)}$ are the charge transfer numbers ($n=m$) and “transition charge transfer numbers” ($n\ne m$) [47] of the diabatic states.

The total singlet and triplet populations were subjected to a kinetic model fit, where the singlet population is split into a “hot” and a “cold” subpopulation:

7 $$\begin{aligned} \frac{\partial }{\partial t} \begin{pmatrix} S_{\mathrm {hot}}(t) \\ S_{\mathrm {cold}}(t) \\ T(t) \end{pmatrix} = \begin{pmatrix} -\frac{1}{\tau _1}-\frac{1}{\tau _c} &{} \quad 0 &{} \quad 0\\ +\frac{1}{\tau _c} &{} \quad -\frac{1}{\tau _2} &{} \quad 0\\ +\frac{1}{\tau _1} &{} \quad +\frac{1}{\tau _2} &{} \quad 0\\ \end{pmatrix} \begin{pmatrix} S_{\mathrm {hot}}(t) \\ S_{\mathrm {cold}}(t) \\ T(t) \end{pmatrix}, \end{aligned}$$

where $\tau _1$ and $\tau _2$ are fast and slow ISC time constants, and $\tau _c$ is the “cooling” time constant. Solving this differential equation system with $S_{\mathrm {hot}}(0)=1$ and computing the sum of $S_{\mathrm {hot}}$ and $S_{\mathrm {cold}}$ gives the following biexponential function:

8 $$\begin{aligned} S_{\mathrm {hot}}(t)+S_{\mathrm {cold}}(t) = R_{\mathrm {fast}}{\mathrm {e}}^{-t/\tau _{\mathrm {fast}}} + (1-R_{\mathrm {fast}}){\mathrm {e}}^{-t/\tau _\mathrm {slow}}, \end{aligned}$$

where the three parameters of the biexponential function are given by $R_{\mathrm {fast}}=(1/\tau _1-1/\tau _2)/(1/\tau _c+1/\tau _1-1/\tau _2)$, $\tau _{\mathrm {fast}}=1/(1/\tau _1+1/\tau _c)$, and $\tau _{\mathrm {slow}}=\tau _2$. This shows that one can always interpret a biexponential decay as a kinetic model where the initial state has a “hot” and a “cold” subpopulation.

Nuclear motion was analyzed in terms of the normal mode coordinates $\{Q_i\}$, using the recently presented coherence analysis technique [23]. We computed three descriptors: (1) the shift of average position (shiftEX), (2) the ratio of coherent motion versus random motion (cohEX), and (3) the change in standard deviation ($\Delta \sigma$). The first parameter measures whether the minima of the excited states are shifted compared to the ground-state minimum. The second parameter describes whether after excitation the trajectories carry out motion coherently (all trajectories with similar period and phase) or randomly. The third parameter provides information whether the shape of the excited-state PESs is different from the ground-state PES, e.g., because the distribution spreads into several near-isoenergetic minima.

## Results and discussion

### Vertical excitations

**Fig. 3:**
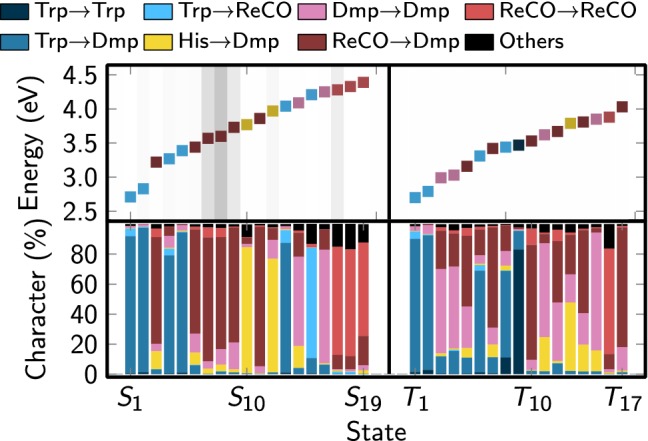
Graphical depiction of the excited-state data given in Table 1. The upper panel shows the excitation energies, oscillator strength (gray background), and state character (color of squares). The lower panel shows the charge transfer contributions to each state

Table 1 compiles the vertical excitation energies, oscillator strengths, charge transfer contributions, and state characters of the 19 excited singlet and 17 triplet states computed at the reference geometry. These states form the diabatic state basis for the definition of the LVC model. The vertical excitation energies in the table are identical to $\epsilon _n$ parameters in Eq. (3). The state characters are assigned based on the charge transfer numbers, where we only show significant contributions and report the remainder as “Others.” The data are graphically presented in Fig. 3.

**Table 1:** Vertical excitation results ($\lambda$: excitation wave length, *E*: excitation energy) for the Trp-[Re(CO)$_3$(Dmp)(His)]$^+$ at the TDA-B3LYP level of theory

State	$\lambda$ (nm)	*E* (eV)	$f_\mathrm {osc}$	Charge transfer numbers (%)									Character^a^
				Hole:	Trp	Trp	Trp	His	Dmp	$\hbox {Re(CO)}_{3}$	$\hbox {Re(CO)}_{3}$	Others	
				Elec.:	Trp	Dmp	$\hbox {Re(CO)}_{3}$	Dmp	Dmp	Dmp	$\hbox {Re(CO)}_{3}$		
$S_{1}$	457	2.71	0.001		0	92	5	0	1	0	0	1	CS
$S_{2}$	438	2.83	0.011		1	96	1	0	1	0	0	0	CS
$S_{3}$	385	3.22	0.003		0	3	0	12	5	71	6	3	MLCT
$S_{4}$	379	3.27	0.011		0	79	4	1	8	6	0	2	CS
$S_{5}$	365	3.39	0.007		1	93	1	1	4	0	0	0	CS
$S_{6}$	360	3.44	0.008		0	6	0	8	13	69	2	2	MLCT
$S_{7}$	347	3.57	0.064		0	0	0	3	5	82	7	2	MLCT
$S_{8}$	344	3.60	0.107		0	2	0	4	10	75	6	3	MLCT
$S_{9}$	332	3.73	0.042		0	1	0	2	18	77	1	1	MLCT
$S_{10}$	329	3.77	0.000		0	1	0	84	2	4	0	9	LLCT
$S_{11}$	321	3.86	0.003		0	0	0	0	5	93	1	1	MLCT
$S_{12}$	312	3.97	0.016		0	1	0	76	13	8	1	2	LLCT
$S_{13}$	307	4.04	0.003		1	86	8	1	2	0	0	1	CS
$S_{14}$	303	4.09	0.002		0	4	0	14	60	16	2	4	IL
$S_{15}$	294	4.21	0.001		0	10	73	0	0	0	2	13	AMCS
$S_{16}$	292	4.25	0.003		0	6	0	1	76	13	0	3	IL
$S_{17}$	289	4.28	0.032		0	0	1	1	1	9	72	15	MC
$S_{18}$	286	4.33	0.001		0	0	1	0	1	9	71	17	MC
$S_{19}$	282	4.39	0.001		0	0	0	2	3	20	62	12	MC
$T_{1}$	459	2.70	–		1	89	5	0	3	0	0	1	CS
$T_{2}$	444	2.79	–		3	90	0	0	6	0	0	0	CS
$T_{3}$	414	2.99	–		0	11	0	2	56	26	2	2	IL
$T_{4}$	409	3.03	–		0	15	1	1	54	22	2	4	IL
$T_{5}$	392	3.16	–		0	11	0	6	28	47	5	3	MLCT
$T_{6}$	374	3.31	–		1	68	4	1	6	17	2	2	CS
$T_{7}$	362	3.42	–		1	11	0	8	11	67	1	1	MLCT
$T_{8}$	360	3.44	–		11	58	0	3	10	17	0	1	CS
$T_{9}$	357	3.47	–		83	12	0	0	1	1	0	2	AC
$T_{10}$	351	3.53	–		0	2	0	0	7	77	11	3	MLCT
$T_{11}$	342	3.62	–		0	2	0	23	62	6	1	6	IL
$T_{12}$	338	3.67	–		0	7	0	1	39	41	8	4	MLCT
$T_{13}$	327	3.79	–		0	2	0	46	19	26	2	6	LLCT
$T_{14}$	325	3.81	–		0	2	0	18	21	55	1	3	MLCT
$T_{15}$	322	3.85	–		0	2	0	14	78	3	1	2	IL
$T_{16}$	319	3.88	–		0	0	0	1	2	10	70	16	MC
$T_{17}$	307	4.03	–		0	1	0	1	16	80	1	1	MLCT

^a^*CS* charge separated, *MLCT* metal-ligand charge transfer, *LLCT* ligand–ligand charge transfer, *IL* intraligand, *AMCS* amino acid–metal charge separated, *AC* amino acid centered, *MC* metal centered

For the Trp-[Re(CO)$_3$(Dmp)(His)]$^+$ system, the lowest singlet and triplet excited states can be found at excitation energies of about 2.7–2.8 eV, based on the TDA-B3LYP calculation. These low-lying states can be identified as charge-separated (CS) states, based on the fact that more than 90% of the states can be described as excitations from Trp orbitals to Dmp orbitals. The lowest non-CS singlet state is $S_3$ at 3.22 eV with MLCT character, and the lowest non-CS triplet state is $T_3$ with IL (intraligand) character. At higher energies, we find a large number of MLCT states, some LLCT states (ligand-ligand charge transfer, in this case His to Dmp), an AMCS state (amino acid–metal CS), several MC states (metal centered), and one amino acid-centered (AC) state. These results agree qualitatively with previously published results [6].

One of the most important properties of the computed states in the present context is the oscillator strength, because it governs which are the initial electronic states after vertical excitation. Here, it is interesting to note that in the low-energy region (around 3 eV) the states with the highest oscillator strengths are the CS states, not the MLCT states. As we will discuss below, this has a profound influence on the initial wave function character in the SHARC–LVC simulations.

### Absorption spectrum

**Fig. 4:**
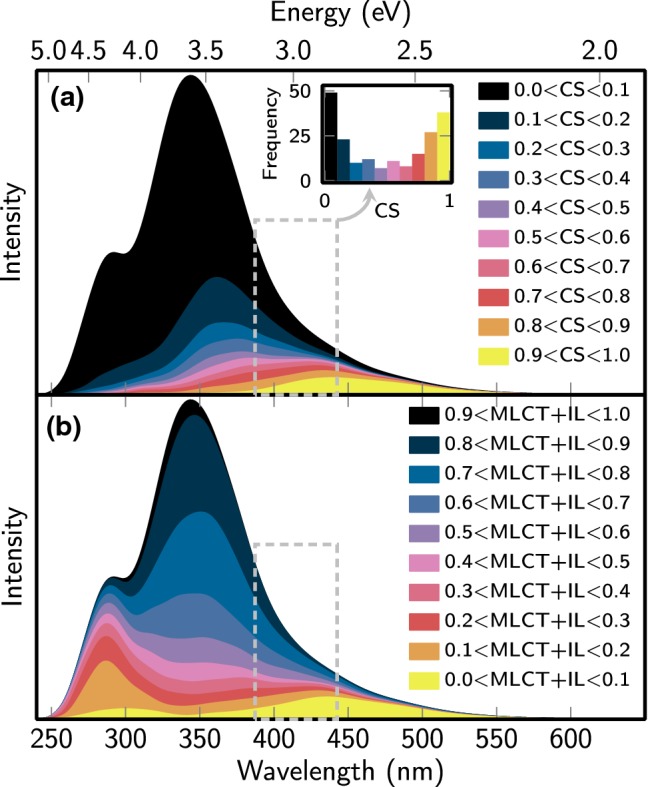
Simulated absorption spectrum and decomposition into different charge transfer contributions. In **a**, we decompose the spectrum into states with low CS contributions (black to blue) and high CS contributions (red to yellow). In **b**, we decompose the spectrum into states with high MLCT and/or IL contributions (black to blue) and low MLCT and/or IL contributions (red to yellow). The dashed boxes indicate the initial excitation window

The previous results indicate that there are low-lying bright CS states available in the system to be excited. However, the trajectories are not started exactly at the reference geometry, but from a Wigner distribution centered on the reference geometry. Hence, it is expedient to analyze how the distribution of geometries due to vibrational motion in the ground state affects the vertical excitation energies, oscillator strengths, and charge transfer characters. This information is presented compactly in the absorption spectrum presented in Fig. 4, obtained by Gaussian convolution (FWHM of 0.2 eV) of the vertical excitation energies and oscillator strengths of all states of all 1000 sampled geometries. The figure also decomposes the spectrum into contributions with different amount of CS character (a) and of MLCT or IL character (b). The dashed boxes indicate the initial excitation window of 2.8–3.2 eV that we employed to select the initial electronic states.

As can be seen, the simulated absorption spectrum has a pronounced absorption tail extending to about 550 nm. Most of this strong absorption tail can be attributed to states with a CS contribution of more than 90%. On the contrary, the very intense band at 340 nm and the shoulder at 290 nm are almost exclusively due to states with very low CS character. Panel (b) of the figure further refines this picture, showing that the high-intensity band is mostly due to states with a combined MLCT + IL contribution of more than 50%, whereas the shoulder at 290 nm (4.3 eV) is due to states with low MLCT + IL character. Further inspection showed that the shoulder is due to MC states (e.g., the diabatic $S_{17}$ shown in Table 1).

The charge transfer character of the low-lying excited states notably affects the character of the states that are excited by our stochastic selection algorithm. As we excite at only 387–443 nm, respectively, 2.8–3.2 eV (gray rectangle), we also excite a significant fraction of the CS states with nonzero oscillator strength. The amount of trajectories excited into states with different CS character inside the excitation window is shown in the inset in Fig. 4a. It can be seen that the largest number of trajectories starts in states with almost zero CS character (49 trajectories start below 10%), but also that the second largest quantile is the states with pure CS character (38 trajectories start above 90%). Naturally, this initial distribution of the CS character will strongly affect the evolution of the electronic wave function.

### Nonadiabatic dynamics

**Fig. 5:**
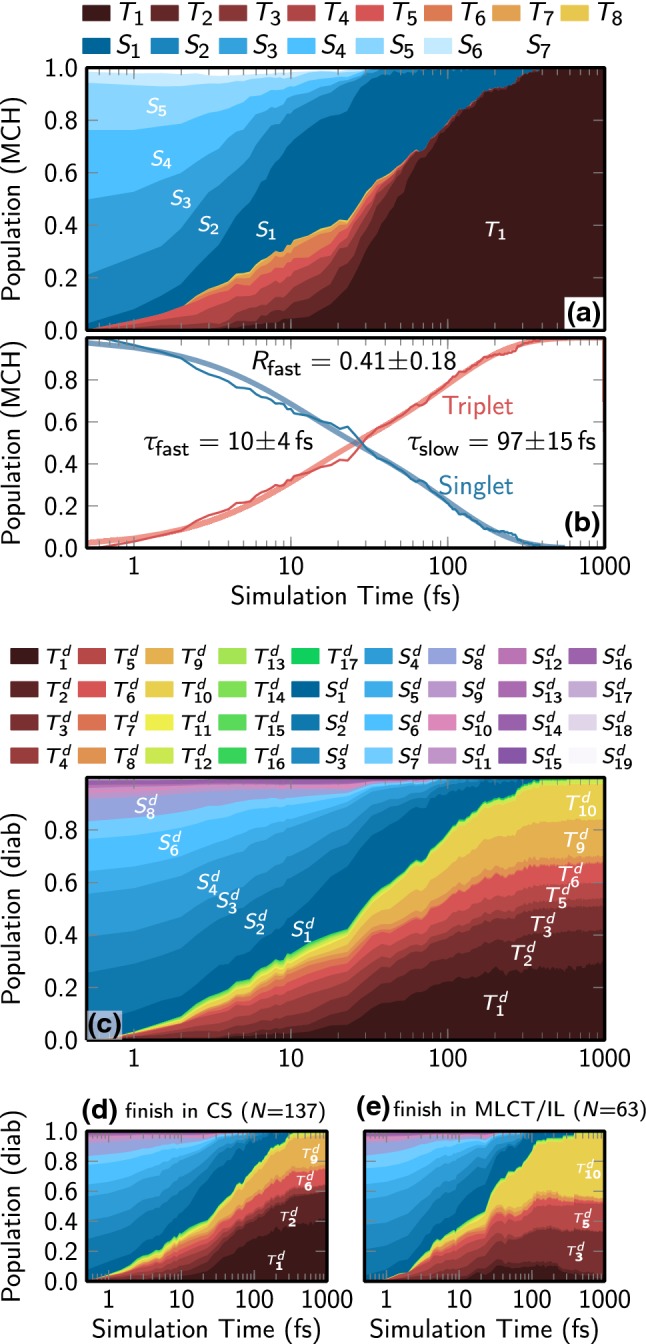
**a** Evolution of the electronic populations in the MCH representation. Note that higher states ($S_8$ to $S_{19}$, $T_9$ to $T_{17}$) are not populated and hence are not shown for clarity. **b** Sum of singlet and triplet states, together with curves fitted with a biexponential decay model. **c** Evolution of the electronic populations in the diabatic representation. Note that the state labels used here refer to the diabatic basis (superscript *d*), as shown in Table 1 and are not identical to the state labels in **a**. The lower panels show the diabatic populations for **d** trajectories finishing in an CS state and **e** trajectories finishing in an MLCT or IL state

The temporal evolution of the MCH populations is shown in Fig. 5 on a logarithmic time scale. As can be seen, initially the electronic wave function is a linear combination of all the singlet states that are available within the excitation window (mostly $S_1$ to $S_5$). However, these singlet states are very short-lived, to the extent that after 20 fs, only $S_1$ is populated besides the triplet states. $S_1$ is fully depleted after 300 fs. This quick decay of the singlet population is matched by the quick rise of the triplet states. In the first 50 fs, ISC drives the population from the singlet states to the triplet manifold, populating $T_1$ to $T_7$ states. The higher-lying triplet states are also rather short-lived, and decay to $T_1$ state within 100 fs.

We fitted the total singlet and triplet population to the biexponential decay model discussed above, as shown in Fig. 5b. We find that, initially, the singlet population decays with a time constant of approximately 10 ± 4 fs. This result is in very good agreement with the 8 fs time constant found previously in SHARC simulations of the “small system” [Re(CO)$_3$(imidazole)(phenanthroline)]$^+$ in water [22]. This very fast ISC process is due to the strong SOCs in the Re complex, which leads to the formation of a spin–orbit wave packet that quickly decoheres to a statistical singlet-triplet mixture [22].

While the fast decay constant is in very good agreement with the simulations in aqueous environment, the slow time constant differs notably (97 fs here, 420 fs in aqueous environment) [22]. In the previous simulations, this time constant was due to nuclear motion that relaxes the energy of the system. Since triplet states are available at lower energies than singlet states, this relaxation lead to a shift of the statistical singlet-triplet mixture to a more pure triplet character [22]. In the presence of the Trp moiety, a pure triplet character is attained much quicker. This is possibly due to the presence of the low-lying triplet CS states. Due to the fact that the CS states are not localized on the metal atom, they exhibit much smaller SOCs than the MLCT states. Hence, once a trajectory has relaxed to a CS minimum, SOCs become weak and the state becomes a pure triplet state. As a consequence, the availability of the low-lying CS states seems to promote a faster decay of the singlet population.

Figure 5c presents the electronic populations transformed into the diabatic basis, i.e., in the basis spanned by the states that are given in Table 1 and Fig. 3. It can be seen that a relatively large number of diabatic states is appreciably populated. In particular, it can be seen that at late simulation times the low-lying MCH triplet state (see Fig. 5a) is composed of seven different diabatic triplet states ($T_1$, $T_2$, $T_3$, $T_5$, $T_6$, $T_9$, $T_{10}$).

Individual inspection of the trajectories revealed that there are two different classes of trajectories, one that finishes in a predominantly CS state, and another one that finishes in a mixture of MLCT and IL states. To gain some further insight into the contributions of the diabatic states, we computed the diabatic populations of these two classes separately, as shown in Fig. 5d, e. As can be seen in panel (d), the CS-dominant trajectories have contributions from $T_1$, $T_2$, $T_6$, and $T_9$ (CS states and an amino acid-centered state). On the contrary, the MLCT-dominated trajectories (panel (e)) have mostly wave function contributions from $T_3$, $T_5$, and $T_{10}$ (IL and MLCT states).

**Fig. 6:**
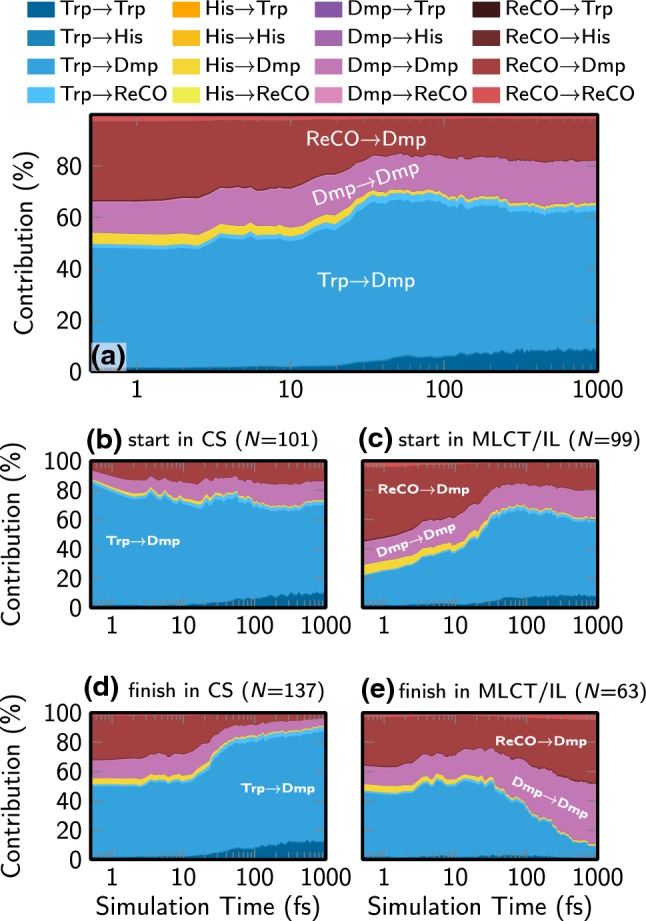
Evolution of the electronic populations in terms of the different charge transfer contributions as a stacked area plot. **a** Full ensemble of 200 trajectories. **b** Only trajectories with more than 50% CS contribution at $t=0$. **c** Only trajectories with less than 50% CS contribution at $t=0$. **d** Only trajectories with more than 50% CS contribution at $t=1000$ fs. **e** Only trajectories with less than 50% CS contribution at $t=1000$ fs. *N* in the labels refers to the number of trajectories

Even though one can draw basic conclusions from the diabatic populations and the state characters of the diabatic basis states, one can get more accurate and objective insight into the electronic evolution from the direct computation of the charge transfer descriptors for the trajectories. Figure 6 presents the corresponding results. In panel (a), we show the evolution for the full ensemble of 200 trajectories. It can be seen that initially, CS character—i.e., Trp $\rightarrow$ Dmp charge transfer—contributes about 50% to the initial states of all trajectories, with the remainder being mostly Dmp $\rightarrow$ Dmp (IL) and Re(CO)$_3\rightarrow$Dmp (MLCT) characters. During the dynamics, the CS contribution first rises briefly to about 60%, before decreasing to a value of about 55% that is reached at 300 fs and kept until the end of the simulation time. At $t=0$, the MLCT contribution is the second largest, with slightly over 30% contribution; IL excitations contribute 10%. In the course of the dynamics, the MLCT contribution quickly decays and from 100 fs, the MLCT and IL contributions are nearly identical. At $t=1000$ fs, MLCT and IL each contribute 16%. Finally, one can observe an increase in amino acid-centered excitations (Trp $\rightarrow$ Trp), reaching 8% at $t=1000$ fs.

From Fig. 6a, one could draw the general conclusion that the system exhibits relatively little dynamical changes, as the charge transfer contributions do not change dramatically. However, one can identify more details on the dynamics from the evolution of the sub-ensembles plotted in Fig. 6b–d. In panel (b), we only include the 101 trajectories whose initial active state was predominantly CS, whereas in panel (c), we analyzed the remaining 99 trajectories. Here, it is interesting to note that both sub-ensembles reach similar compositions at $t=1000$ fs. This seems to indicate that there is some charge transfer dynamics that is hidden in panel (a), i.e., trajectories are not trapped in their initial state but can and do switch between CS and MLCT/IL. This conclusion is reinforced by panels (d) and (e), which show that trajectories finishing with CS or MLCT/IL character all start from nearly indistinguishable starting compositions.

At this point, it is expedient to discuss our results with respect to the most relevant literature findings. Here, especially the detailed discussion of the photocycle (Fig. 2) by Blanco-Rodriguez et al. [13] is most useful. One of the conclusions drawn by them is that due to the life time of $^{1}\hbox {MLCT}$ state of about 110 fs, there is sufficient time for electron transfer leading to the population of $^{1}\hbox {CS}$ states, which subsequently converts to $^{3}\hbox {CS}$ via ISC. On the contrary, they ruled out that $^{1}\hbox {CS}$ state is directly excited by absorption, due to the negligible oscillator strengths found in their TDDFT calculations. Both statements—about the role of $^{1}\hbox {MLCT}\rightarrow {} ^{1}\hbox {CS}\rightarrow {} ^{3}\hbox {CS}$ route and about the role of direct excitation of $^{1}\hbox {CS}$ states—might need to be revised based on our results.

First, all nonadiabatic dynamics simulations published so far [17–20, 22] and the present results strongly indicate that the lifetime of a pure $^{1}\hbox {MLCT}$ is on the order of only 10 fs, after which a spin-mixed $^{1,3}\hbox {MLCT}$ wave function is formed due to the large SOCs. Hence, it is very unlikely that the $^{1}\hbox {MLCT}\rightarrow {} ^{1}\hbox {CS}$ pathway is of high relevance. Rather, one can expect that the spin-mixed $^{1,3}\hbox {MLCT}$ wave function will evolve into a linear combination of $^{1}\hbox {CS}$ and $^{3}\hbox {CS}$, which later might collapse into either multiplicity. Stated differently, we expect a complicated mechanism where ET and ISC occur simultaneously.

Second, the computations of Marazzi et al. [6] and our results indicate that the low-lying $^{1}\hbox {CS}$ states can acquire a non-negligible oscillator strength. Even though the oscillator strengths are quite small (all CS states have oscillator strength $\le 0.01$), they are still relevant because at the red tail of the absorption band (below 3 eV) there are no other states with larger oscillator strength. The first bright $^{1}\hbox {MLCT}$ states occur around 3.6 eV (see Table 1), in accordance with previous results [16] that showed that the torsion angle of the imidazole (His) group determines the energy of the lowest MLCT state. Hence, excitation at energies around 3.0 eV has a non-negligible chance of exciting a state with significant CS character.

**Fig. 7:**
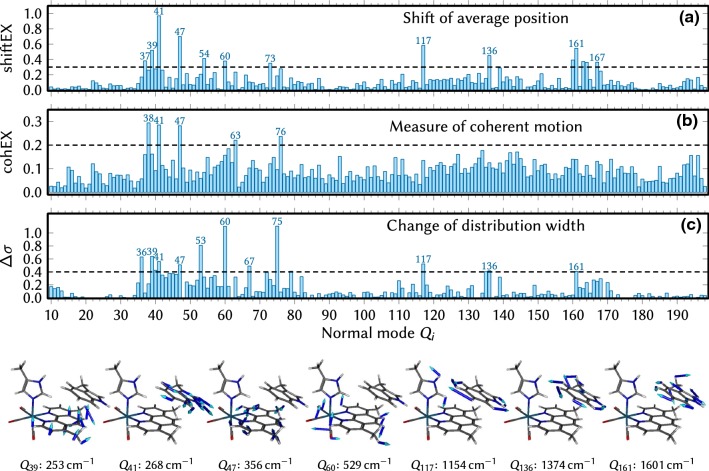
Normal mode coherence analysis [23] of the nuclear motion in the SHARC trajectories. **a** The shiftEX parameter describes how much the average position of the ensemble shifts after the excitation. **b** The cohEX parameter describing the extent of coherent, in-phase motion. **c**$\Delta \sigma$ parameter measures how much the distribution broadens after excitation. The pictures below show the 7 normal modes that are marked at least twice in **a**–**c**

The results of the normal mode coherence analysis are shown in Fig. 7. The three parameters measure different aspects of the nuclear motion, as described above. Based on these results, we define the most important modes to be the ones that are above the dashed thresholds in at least two panels. In this way, we obtain a set of 7 most important normal modes: $Q_{39}$, $Q_{41}$, $Q_{47}$, $Q_{60}$, $Q_{117}$, $Q_{136}$, and $Q_{161}$. These modes are depicted in the bottom part of Fig. 7, showing that all modes either involve in-plane vibrations of the Trp moiety, vibrations of the Dmp ligand, or Re–C=O bending. The latter two types of normal modes were already observed to be important [23] in the ab initio dynamics simulations of [Re(CO)$_3$(Phen)(Im)]$^+$, where it was found that the MLCT states are most strongly coupled via in-plane vibrations of Phen and modes involving the Re–C=O bonds. It seems natural that here in the dynamics including the Trp moiety, we additionally find normal modes corresponding to vibrations of Trp.

### Limitations

At this point, a brief discussion of the limitations of the presented simulations is in order. The first aspect of the simulations worth mentioning is the LVC model of the PESs that is the basis of our nonadiabatic dynamics simulations. This model is based on the vertically and horizontally shifted harmonic oscillator for the ground-state minimum. While this approximation should be reasonable in the vicinity of the reference geometry (ground-state minimum), it can be expected that the accuracy of the PESs deteriorates when one moves away from the reference point; for example, many of the low-frequency normal modes in the model will probably lead to significantly anharmonic potentials, which cannot be described by the LVC approach. Furthermore, the curvature of the excited-state potentials is likely to be different from the one of the ground-state potential. However, it is unclear how severe the latter approximation is, as LVC models worked well for the [Re(CO)$_3$(Phen)(Im)]$^+$ complex without Trp [17–20].

The second limiting aspect that deserves mention is the electronic structure level of theory. We are aware that especially the choice of the exchange-correlation functional could have a profound impact on the obtained PESs. Here, it is especially important that the functional is able to accurately describe the energies of long-range charge transfer states, relative to the energies of local excitations. Although in general, long-range charge transfer is better described by functionals with a high admixture of Hartree–Fock exchange or by range-separated hybrid functions [55], previously reported computations on the [Re(CO)$_3$(Phen)(Im)]$^+$ plus Trp system [6, 13] showed that this system is reasonably well described with standard hybrid functionals like PBE0 or B3LYP.

A third approximation employed implicitly here is the fact that we model the geometrical constraint of the protein simply by the fixed distance between the two carbon atoms of the methyl groups that replace the protein backbone. However, it is likely that the protein constrains the system further, i.e., it leads to a predominant orientation of the methyl–imidazole and methyl–indole bonds, a fact that is not present in our calculations. This lack of rigidity is at least partially canceled in our simulations, because the employed normal mode coordinate system cannot describe efficiently torsions or rotations of groups of atoms, such that the relative orientation of Dmp and Trp is preserved in our simulations.

## Conclusions

In the present study, we investigated the ultrafast nonadiabatic dynamics of the complex [Re(CO)$_3$(Dmp)(His)]$^+$ (Dmp $=$ 4,7-dimethylphenanthroline) attached to an azurin protein and in close proximity to a Trp side chain of that protein. It was previously found experimentally [9, 13] that after photoexcitation two distinct electronic states are populated on a sub-ps time scale. These two states are a triplet metal-to-ligand charge transfer ($^{3}\hbox {MLCT}$) state and a triplet charge-separated ($^{3}\hbox {CS}$) state where an electron is transferred from Trp to Dmp. The results of our TDDFT calculations, charge transfer analysis, and nonadiabatic dynamics simulations using the SHARC-LVC method indicate that there are several pathways that lead to the population of these two electronic states. First, photoexcitation can directly populate both singlet MLCT and singlet CS states, as both of these exhibit nonzero oscillator strength depending on geometry. After excitation, a complicated mechanism involving parallel and simultaneous intersystem crossing and electron transfer occurs, which leads to the establishment of a pre-equilibrium between $^{3}\hbox {MLCT}$ and $^{3}\hbox {CS}$ states. These results confirm the assignment of the sub-ps time constants of the related experiments [9, 13] and constitute the first computational evidence for the ultrafast formation of the charge-separated states in Re-sensitized azurin. Similar competitive intersystem crossing and electron transfer phenomena have been observed in other protein-coupled transition metal complexes [56–58], for example in Re(CO)$_3$ diimine complexes anchored to different locations in azurins [8, 12], Re(CO)$_3$ diimine complexes in azurins with two mediating tryptophan moieties [59], or Ru complexes in azurins [60]. The study of these competing photoinduced processes is critical in understanding and utilizing electron transfer through proteins [57, 58].
